# Isolation and characterization of the dimetal decacarbonyl dication [Ru_2_(CO)_10_]^2+^ and the metal-only Lewis-pair [Ag{Ru(CO)_5_}_2_]^+^ †

**DOI:** 10.1039/d4dt03364g

**Published:** 2024-12-06

**Authors:** Malte Sellin, Jörg Grunenberg, Ingo Krossing

**Affiliations:** a Institut für Anorganische und Analytische Chemie und Freiburger Materialforschungszentrum (FMF), Albert-Ludwigs-Universität Freiburg Albertstr. 21 79104 Freiburg Germany krossing@uni-freiburg.de; b Institut für Organische Chemie; Technische Universität Braunschweig Hagenring 30 38106 Braunschweig Germany

## Abstract

The reaction of Ag^+^ with Ru_3_(CO)_12_ in a CO atmosphere under concommittant irradiation with UV-light yields a salt of the metal-only Lewis-pair [Ag{Ru(CO)_5_}_2_]^+^. Switching the silver cation for a more process-selective deelectronator yields a salt of the homoleptic transition metal carbonyl cation [Ru_2_(CO)_10_]^2+^, which fills the gap between the known cations [Ru(CO)_6_]^2+^ and [Ru_3_(CO)_14_]^2+^. The amount of π-backdonation in this series was studied by a combination of vibrational spectroscopy and computed relaxed force constants.

## Abstract 2

Upon irradiation and carbon monoxide pressure, ruthenium carbonyl reacts with silver(i) and deelectronating reagents to form [Ag{Ru(CO)_5_}_2_]^+^ and [Ru_2_(CO)_10_]^2+^, respectively.

## Graphical abstract

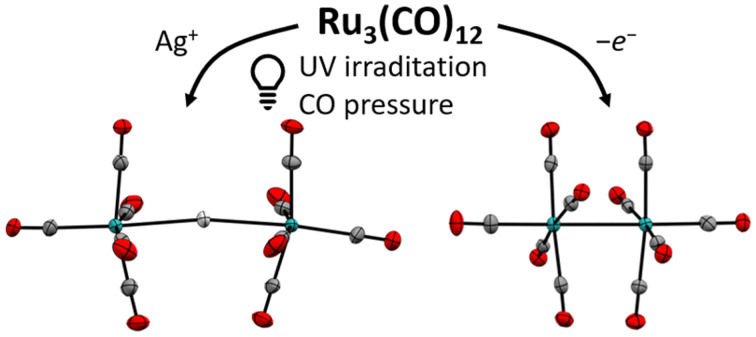

## Introduction

Carbon monoxide (CO) is an excellent π-acceptor and a moderate σ-donor ligand. With this combination, transition metal carbonyls (TMCs) are amongst the most fundamental complex classes in organometallic chemistry.^1,2^ Their electronic properties are ideal for the stabilisation of unusually low formal oxidation states of transition metals down to −IV.^3^

By contrast, the stabilization of metal cations exclusively by carbon monoxide ligands needs the strict absence of nucleophiles. Therefore, the first homoleptic transition metal carbonyl cation (TMCC) – [Mn(CO)_6_]^+^ – was not isolated until 1961 by Fischer *et al.*^4^ Even after that, little progress on this kind of compounds was made until the 1990s, when the groups of Willner and Aubke introduced superacidic systems such as HF/SbF_5_ or FSO_3_H/SbF_5_ to isolate various closed-shell TMCCs up to the oxidation state + III.^5–7^ Most of these TMCCs are “non-classical” carbonyl complexes in which σ-donation is stronger than π-backdonation, yielding positively polarized (non-classical) carbonyl ligands, which have a higher force-constant than uncoordinated carbon monoxide.^2,6,8^ ‡‡We use the subdivision of (non-)classical not for the entire complex, but separated for the interaction between the transition metal and each spectroscopically independent carbonyl ligand.^9,14^

Over the last years, our group prepared^9^ several missing TMCCs by the reaction of binary TMCs with deelectronators (also known as one-electron oxidants)§§The deelectronation is a special-case of the oxidation reaction and describes ‘a complete net removal of one or more electrons from a molecular entity’ (https://doi.org/10.1351/goldbook.O04362).^53^ such as [NO]^+^, Ag^+^ or synergistic Ag^+^/0.5 I_2_ yielding the open-shell TMCCs [Ni(CO)_4_]˙^+^,^10^ [M(CO)_6_]˙^+^ (M = Cr, Mo, W)^11^ and the heptacarbonyls [M(CO)_7_]^+^ (M = Nb, Ta).^12^ However, often other oxidation paths (*e.g.* halonium addition) were observed besides the desired deelectronation.§ Thus, we developed selective deelectronation reagents first on the basis of fluorinated ammoniumyl,^13^ then aromatic radical cations,^14–17^*e.g.* [anthracene^Hal^]˙^+^ (anthracene^Hal^ = C_14_F_8_Cl_2_) with a formal potential of +1.42 V *vs.* ferrocene. Using these reagents, even strongly Lewis-basic TMCs like W(CO)_6_ and Fe(CO)_5_ can be selectively deelectronated and isolated as their respective radical-cation salts.^15,18,19^

**Fig. 1:**
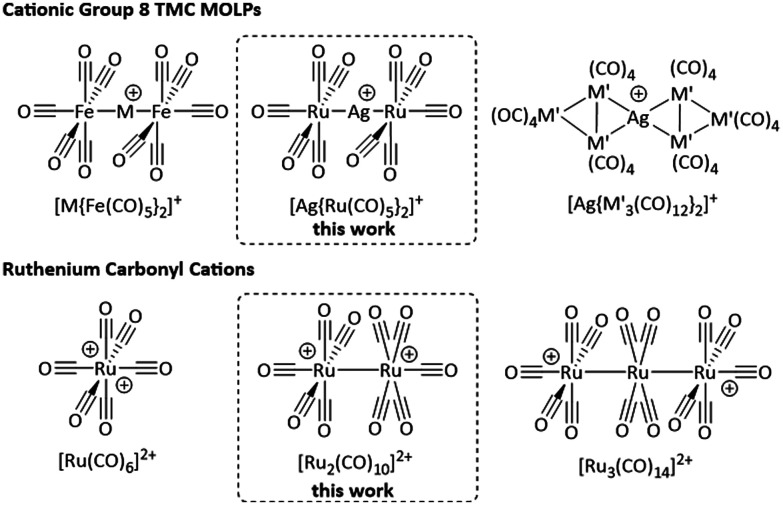
Overview to the cationic group 8 TMC metal-only Lewis pairs (MOLPs; M = Cu, Ag, Au; M′ = Ru, Os) and the ruthenium carbonyl cations in comparison to the MOLP based TMCC [Ag{Ru(CO)_5_}_2_]^+^ and the homodinuclear TMCC [Ru_2_(CO)_10_]^2+^ from this work.

Attempts to isolate the heavier homologues of the radical cation [Fe(CO)_5_]˙^+^ by the reaction of the respective trimetal dodecacarbonyls with [anthracene^Hal^]˙^+^ under carbon monoxide atmosphere yielded the clustered TMCCs [M_3_(CO)_14_]^2+^ (M = Ru, Os) instead.^14^ These complexes are members of the even smaller family of homomultinuclear TMCCs, with the only other examples being [Pt_2_(CO)_6_]^2+^ ^20^ and [Hg_2_(CO)_2_]^2+^.^21^ By contrast, a series of heteromultinuclear TMCCs forms by combining TMCs with coinage metals and yields the class of metal-only Lewis pair (MOLP)^22^ cations [M{TMC}_2_]^+^, *e.g.* [M{Fe(CO)_5_}_2_]^+^, [Ag{M′_3_(CO)_12_}_2_]^+^ (M = Cu, Ag, Au; M′ = Ru, Os; Fig. 1).^14,23–27^

## Results and discussion

Herein, we report the light-induced reaction of the cluster Ru_3_(CO)_12_ to the metal-only Lewis-pair [Ag{Ru(CO)_5_}_2_]^+^ and the all-Ru^I^ transition metal carbonyl cation [Ru_2_(CO)_10_]^2+^, filling the gap between superelectrophilic [Ru(CO)_6_]^2+^ and the [Ru_3_(CO)_14_]^2+^ cluster.^14,28^

### Synthesis and characterisation of [Ag{Ru(CO)_5_}_2_]^+^

We previously reported that the silver(i) cation reacts with Ru_3_(CO)_12_ as a Lewis-acid instead as an oxidant and forms [Ag{Ru_3_(CO)_12_}_2_]^+^.^14^ Repeating the same reaction with an excess of silver(i) under irradiation with UV-light (370 nm) and carbon monoxide pressure (3 bar), the silver(i) cation still did not react as an oxidation reagent, but rather formed [Ag{Ru(CO)_5_}_2_]^+^ (eqn (1); [F{Al(OR^F^)_3_}_2_]^−^ counterion).1
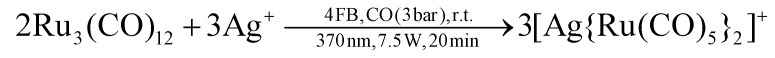


**Fig. 2:**
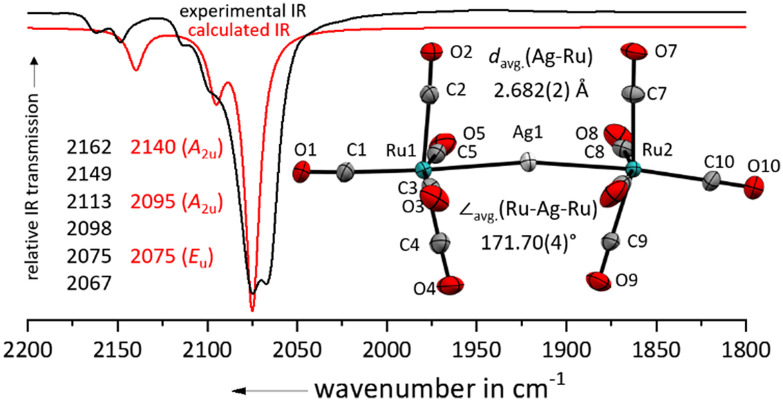
Experimental ATR-IR spectrum of [Ag{Ru(CO)_5_}_2_]^+^[F{Al(OR^F^)_3_}_2_]^−^ (black line) in comparison with the and calculated IR spectrum of [Ag{Ru(CO)_5_}_2_]^+^ (red line, B3LYP(D3-BJ)/def2-TZVPP, scaled by 0.968 according to Duncan *et al.*^31^). Note the doubling of all lines by symmetry lowering induced by bending. Solid-state structure of the complex cation in [Ru_2_(CO)_10_]^2+^([F{Al(OR^F^)_3_}_2_]^−^)_2_·(4FB)_2_. Ellipsoids depicted at 50% probability; colour code: silver – light grey, ruthenium – turquoise, oxygen – red, carbon – light grey.

Layering the colourless 4FB solution (4FB = 1,2,3,4-tetrafluorobenzene) with *n*-pentane led to the formation of a 89% yield of colourless plates suitable for single crystal X-ray diffraction studies of [Ag{Ru(CO)_5_}_2_]^+^[F{Al(OR^F^)_3_}_2_]^−^ (R^F^ = C(CF_3_)_3_; Fig. 2). Related to complexes between iron pentacarbonyl and metal cations [M{Fe(CO)_5_}_2_]^*x*+^ (M = Cu^+^, Ag^+^, Au^+^, Hg^2+^), the silver(i) cation binds directly to the metal centre of the pentacarbonyl complex.^24–27,29^ In comparison to the nearly linear Fe–M–Fe cores, the Ru–Ag–Ru axis is with 171.7° significantly bent, which could be a consequence of weaker secondary Ag⋯C contacts due to the larger Ag–Ru distance. Still, the Ag–Ru distances are with 2.682(2) Å in [Ag{Ru(CO)_5_}_2_]^+^ significantly shorter than in [Ag{Ru_3_(CO)_12_}_2_]^+^ (2.887(1) Å).^14^ This bending also leads to a lowering of the symmetry and a splitting/doubling of all IR bands in comparison to the optimized *D*_4h_ symmetric structure.

We may speculate that the [Ag{Ru(CO)_5_}_2_]^+^ cation could serve in the future as a synthon for the highly unstable Ru(CO)_5_, as the Fe(CO)_5_ in the closely related [Ag{Fe(CO)_5_}_2_]^+^ can be transmetalated to copper using CuBr.^29^

### Synthesis and characterisation of [Ru_2_(CO)_10_]^2+^

Since we aimed for a homonuclear TMCC, we switched to halogenated arenium radical cations as more selective deelectronators. Initial attempts to generate the [Ru_2_(CO)_10_]^2+^ dication by the reaction of Ru_3_(CO)_12_¶¶Monomeric Ru(CO)_5_ was not used, because it is only obtainable by a high-pressure carbonylation of ruthenium or Ru_3_(CO)_12_. Due to its rapid loss of CO to form Ru_2_(CO)_9_ and finally Ru_3_(CO)_12_, it is not commercially available.^54^ with an excess of the deelectronator [anthracene^Hal^]˙^+^ under carbon monoxide atmosphere only yielded [Ru_3_(CO)_14_]^2+^, unreacted [anthracene^Hal^]˙^+^ and [Ru_2_(CO)_10_]^2+^ as a side-product. Even exchanging [anthracene^Hal^]˙^+^ for the far more powerful [naphthalene^F^]˙^+^ deelectronation reagent (naphthalene^F^ = C_10_F_8_; +2.00 V *vs.* Fc^+/0^)^15,16^ led to the same result.

However, when a 4FB solution of Ru_3_(CO)_12_ and [anthracene^Hal^]˙^+^[WCA]^−^ (WCA = [Al(OR^F^)_4_]^−^, [F{Al(OR^F^)_3_}_2_]^−^) is irradiated with UV-light (370 nm) under carbon monoxide pressure (3 bar), the respective [Ru_2_(CO)_10_]^2+^([WCA]^−^)_2_ complex salts forms as a major product besides some minor unknown side-products in 66% overall yield (eqn (2); [Al(OR^F^)_4_]^−^/[F{Al(OR^F^)_3_}_2_]^−^ counterion).||||Using an excess of Ru_3_(CO)_12_ under the same conditions unfortunately does not yield higher clusters such as [Ru_4_(CO)_18_]^2+^, but [Ru_3_(CO)_14_]^2+^ and polymeric {Ru(CO)_4_}.2
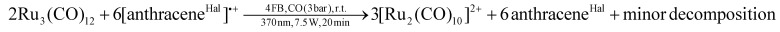


**Fig. 3:**
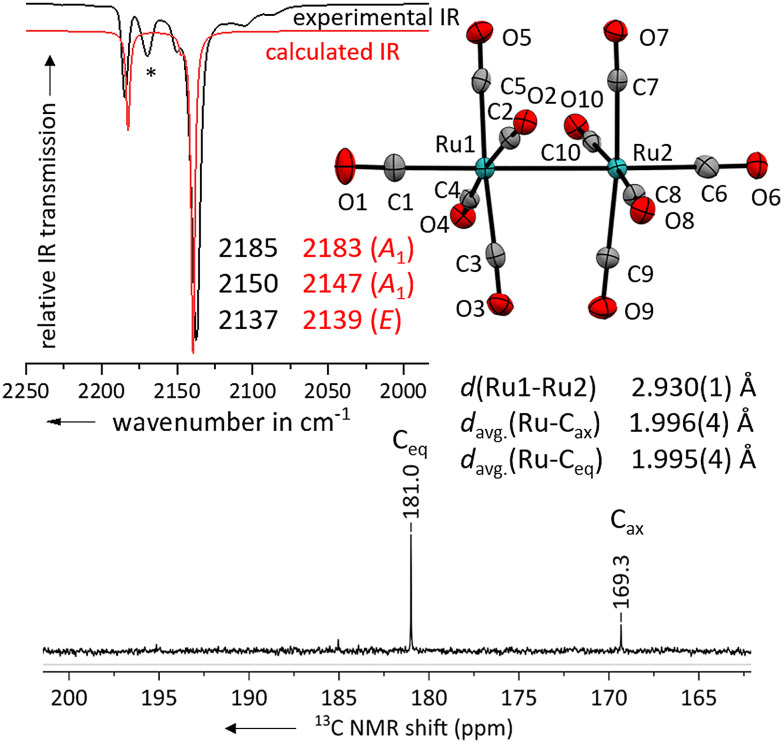
Experimental ATR-IR spectrum of [Ru_2_(CO)_10_]^2+^([Al(OR^F^)_4_]^−^)_2_ (black line, impurity marked with (*)) in comparison with the calculated IR spectrum of [Ru_2_(CO)_10_]^2+^ (red line, B3LYP(D3-BJ)/def2-TZVPP, scaled by 0.968 according to Duncan *et al.*^31^). Solid-state structure of the complex cation in [Ru_2_(CO)_10_]^2+^([F{Al(OR^F^)_3_}_2_]^−^)_2_·(4FB)_2_. Ellipsoids depicted at 50% probability; colour code: ruthenium – turquoise, oxygen – red, carbon – grey. Carbonyl region of the ^13^C NMR spectrum of [Ru_2_(CO)_10_]^2+^([F{Al(OR^F^)_3_}_2_]^−^)_2_ in 4FB.

Layering the solution with *n*-pentane led to the formation of colourless needles suitable for single crystal X-ray diffraction studies. The solid-state structure of the salt [Ru_2_(CO)_10_]^2+^([F{Al(OR^F^)_3_}_2_]^−^)_2_·(4FB)_2_ shows, that the geometry of the complex dication is close to the expected *D*_4d_ symmetry (Fig. 3) with a torsion angle between the two sets of equatorial carbonyl ligands of only 36° compared to the ideal 45°.

The Ru–Ru bond distance of [Ru_2_(CO)_10_]^2+^ is with 2.930(1) Å slightly longer than the one in the [Ru_3_(CO)_14_]^2+^ cluster with 2.891(1) Å and that in neutral Ru_3_(CO)_12_ at 2.844(2) Å.^30^ The average C≡O distances of the axial carbonyl ligands are to some extent longer than the ones of the equatorial carbonyl ligands. However, as the elongation of the C≡O bond upon π-backdonation is comparably low, this effect is not significant in relation to the higher errors in the scXRD.

To further investigate the electronic properties of the carbonyl ligands, we measured a ^13^C NMR spectrum of [Ru_2_(CO)_10_]^2+^ ([F{Al(OR^F^)_3_}_2_]^−^)_2_ in 4FB. The shift of the ^13^C peak is well suited to compare the electronic situation between different carbonyl ligands bound to the same transition metal, but not in between TMCCs having different transition metals.^9^ The peak for the axial carbonyl ligands is with 169.3 ppm already close to the one observed in [Ru(CO)_6_]^2+^ (168.8 ppm).^7^ This indicates, that the axial carbonyl ligands in [Ru_2_(CO)_10_]^2+^ are positively polarized (non-classical). The equatorial carbonyl ligands are more electron-rich with a ^13^C NMR shift of 181.0 ppm and electronically in-between the axial and equatorial carbonyl ligands of [Ru_3_(CO)_14_]^2+^ (171.6 & 185.1 ppm).^14^

The three bands assigned to the CO-stretching vibrations of [Ru_2_(CO)_10_]^2+^ are in excellent agreement to the DFT-calculated values (B3LYP(D3-BJ)/def2-TZVPP, scaled by 0.968 according to Duncan *et al.*,^31^Fig. 3). Unfortunately, no Raman spectrum could be obtained due to the strong fluorescence induced by minor (photo-)decomposition products.

### Computational investigations on [Ru_2_(CO)_10_]^2+^

**Fig. 4:**
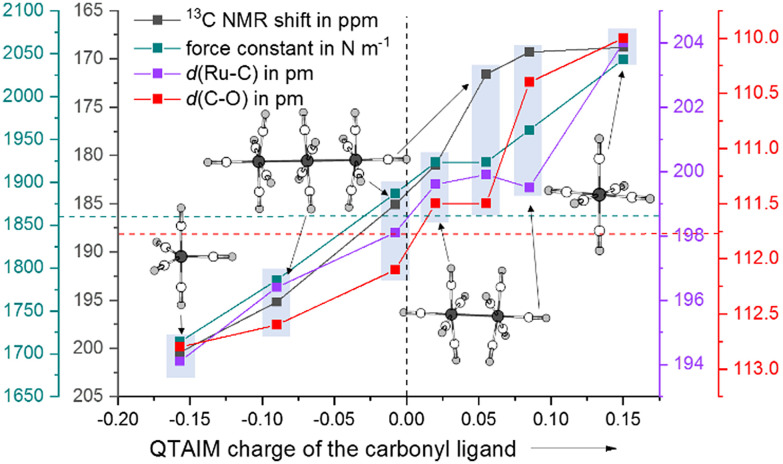
Comparison of the experimental data and calculated force-constants of the carbonyl ligands of Ru(CO)_5_, [Ru_3_(CO)_14_]^2+^, [Ru_2_(CO)_10_]^2+^ and [Ru(CO)_6_]^2+^ as a function on their QTAIM charges. The dotted lines are showing the data of free carbon monoxide.

As a computational measure, which directly distinguishes between classical and non-classical interactions of carbonyl ligands with the metal, we calculated the QTAIM charges (B3LYP(D3-BJ)/def2-TZVPP) of Ru(CO)_5_ and the ruthenium carbonyl cations. The QTAIM charges are in line with the ^13^C NMR shifts throughout the entire series from Ru(CO)_5_ to [Ru(CO)_6_]^2+^: with the increasing positive partial charge of the carbonyl ligand, the ^13^C NMR shift is more high-field. Both the equatorial and axial carbonyl ligands are positively charged, thus making the complex a non-classical TMCC. As already observed in the [Ru_3_(CO)_14_]^2+^ cluster, the interaction of the metal centre with the axial carbonyl ligands are less classical than the equatorial ones (decrease of the π-backdonation). In contrast to the relatively small differences of the ^13^C NMR shifts of the axial carbonyl ligand of [Ru_2_(CO)_10_]^2+^, the carbonyl ligands in [Ru(CO)_6_]^2+^ are nearly twice as positively charged (Fig. 4 and Table 1).

**Table 1:** Comparison of the key spectroscopic/calculated data between [Ru_3_(CO)_14_]^2+^, [Ru_2_(CO)_10_]^2+^ and [Ru(CO)_6_]^2+^

	[Ru_3_(CO)_14_]^2+^	[Ru_2_(CO)_10_]^2+^	[Ru(CO)_6_]^2+^
*ṽ*(CO) (IR) in cm^−1^	2173	2185	2198
	2139	2150	
	2114	2137	
	2058		
*δ* ^13^C_CO_ in ppm	171.5 (ax)	169.3 (ax)	168.8
	185.0 (eq)	181.0 (eq)	
	195.1 (cen)		
QTAIM charge	+0.055 (ax)	+0.085 (ax)	+0.150
	−0.008 (eq)	+0.020 (eq)	
	−0.090 (cen)		
*k*_CO_ in N m^−1^	1.923 (ax)	1.961 (ax)	2.043
	1.887 (eq)	1.923 (eq)	
	1.786 (cen)		

As we were not able to collect a Raman spectrum of the [Ru_2_(CO)_10_]^2+^ complex, the determination of the force-constants from the experimental data would have been inaccurate and would have relied on multiple assumptions. Therefore, we instead computationally determined the full set of relaxed force-constants for the ruthenium carbonyl series using the TPSSH/def2-TZVPP level of theory. Since the values of complete matrix of relaxed force constants do not depend on both, the chosen coordinate system and any assumption concerning the coupling terms, they can be directly used as unique bond strength descriptors.^32^ As expected, both the equatorial and the axial sets of carbonyl ligands in [Ru_2_(CO)_10_]^2+^ have force constants (1.923 & 1.961 N m^−1^) higher than the one of uncoordinated carbon monoxide (1.856 N m^−1^).

### Energetics of the dimerization of [Ru(CO)_5_]˙^+^ to [Ru_2_(CO)_10_]^2+^

While the [Ru_2_(CO)_10_]^2+^ dimer is expected to be preferred against the monomeric 17 VE radical cation [Ru(CO)_5_]˙^+^ according to the isolobal principle, this result stands in contrast to its lighter homologue [Fe(CO)_5_]˙^+^,^18^ which exists as the open-shell monomer under the same conditions. In agreement with this observation, DFT-calculations (B3LYP(D3-BJ)/def2-TZVPP) show, that the dimerization of [M(CO)_5_]˙^+^ to [M_2_(CO)_10_]^2+^ is strongly endergonic in the gas-phase for both iron and ruthenium (Δ_r,(g)_*G*° = + 289.0 kJ mol^−1^ (Fe)/+171.6 kJ mol^−1^ (Ru)) due to the high Coulomb repulsion between the cationic monomers. However, the situation is very different, when the solvation of 4FB is considered, giving Δ_r,(4FB)_*G*° = +101.8 kJ mol^−1^ (Fe)/−9.8 kJ mol^−1^ (Ru) upon inclusion of a COSMO-RS solvation model,^33^ explaining both the open-shell nature of [Fe(CO)_5_]˙^+^ and the dimeric form of [Ru_2_(CO)_10_]^2+^ (eqn (3)).3a
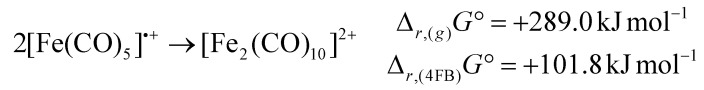
3b
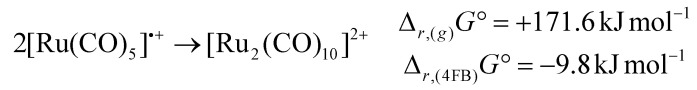


## Conclusions

In summary, we report the isolation and characterization of the MOLP-based TMCC [Ag{Ru(CO)_5_}_2_]^+^ through the reaction of Ru_3_(CO)_12_ and Ag(i) under CO pressure and irradiation with UV-light. Switching the silver(i) cation in this reaction for [anthracene^Hal^]˙^+^, the TMCC [Ru_2_(CO)_10_]^2+^ forms. This complex is the missing link between [Ru(CO)_6_]^2+^ and [Ru_3_(CO)_14_]^2+^. Additionally, this complex can be seen as the dimerized heavier homologue of the recently prepared [Fe(CO)_5_]˙^+^.^18^ Both experimental and quantum-chemical investigations show, that the axial carbonyl ligands are almost as electron-poor as the carbonyl ligands in the superelectrophilic [Ru(CO)_6_]^2+^ and the relevant trends of this new series are plotted in Fig. 4.

## Experimental

### General prodedures

All manipulations were carried out by using standard Schlenk technique or a nitrogen filled glovebox (O_2_/H_2_O < 0.1 ppm). All the reactions were performed in Schlenk tubes with grease free PTFE-valves. The solvent 1,2,3,4-tetrafluorobenzene (4FB, C_6_F_4_H_2_, from fluorochem) was stirred a few days over calcium hydride (CaH_2_) and distilled. The distillate was stirred over Ag^+^[Al(OR^F^)_4_]^−^ and condensed to remove traces of less fluorinated benzenes. This leads to a minor contamination of R^F^OH (≪1%), which does not affect the reactions. *n*-Pentane was dried using a Grubbs apparatus. 4FB, and *n*-pentane were stored over 3 Å molar sieves. Octafluoronaphthalene (ABCR), 9,10-dichlorooctafluoroanthrace (Sigma Aldrich) and triruthenium dodecacarbonyl (Sigma Aldrich) were bought from commercial sources. [NO]^+^[Al(OR^F^)_4_]^−^,^34^ Ag^+^[F{Al(OR^F^)_3_}_2_]^−^, [NO]^+^[F{Al(OR^F^)_3_}_2_]^−^^35^ and [naphthalene^F^]^+^·[F{Al(OR^F^)_3_}_2_]^−^^15^ were prepared according to literature procedures.

### Vibrational spectroscopy

FTIR spectra were recorded inside a glovebox with a Bruker ALPHA equipped with QuickSnap Eco ATR module and ZnSe crystal. The spectra were measured at RT in the range of 4000–550 cm^−1^ with 32 scans and a resolution of 4 cm^−1^. The data were processed with the Bruker OPUS 7.5 software package. The intensities are reported as follows: ≥0.8 = very strong (vs), ≥0.6 = strong (s), ≥0.4 = medium (m), ≥0.2 = weak (w), <0.2 = very weak (vw). The data were processed with the Bruker OPUS 7.5 software package. The graphical representations were created with OriginPro 2021.

### NMR spectroscopy

NMR spectra were recorded at RT on a Bruker Avance DPX 200 MHz. The samples were dissolved in 4FB (0.6 ml) in a 5 mm thick-walled NMR tube with J. Young PTFE valve. The spectra were calibrated by using the ^1^H signal of the solvent 4FB (*δ* = 6.97 ppm, rel. to tetramethylsilane). The field corrections of other nuclei were adjusted accordingly. The MestReNova software package was used for measuring, processing and creation of the graphical representations of the spectra. ^1^H and ^13^C NMR spectra are referenced against TMS and ^19^F NMR spectra against CFCl_3_.

### Single crystal X-ray diffraction

The data were collected on a Bruker D8 VENTURE dual wavelength Mo/Cu three-circle diffractometer with a microfocus sealed X-ray tube using mirror optics as monochromator and a Bruker PHOTON III detector. Single crystals were selected at RT in PFPE oil JC 1800 (Sunoit Performance Material Science), mounted on CryoLoops with a diameter of 0.1 to 0.2 mm and shock-cooled using an Oxford Cryostream 800 low temperature device. The data were gathered at 100(2) K using Mo K_α_ radiation (*λ* = 0.71073 Å). All data were integrated with SAINT (version 8.38A) and a multi-scan absorption correction using SADABS or TWINABS was applied. The structures were solved by direct methods using SHELXT^36^ and refined by full-matrix least-squares methods against F^2^ by SHELXL-2018/3^37^ using the GUI software ShelXle.^38^ Disordered moieties were refined using bond lengths restraints and displacement parameter restraints and were modelled with the program DSR.^39^ The gathered data were finalized with the tool FinalCif.^40^ The graphical representations of the crystal structures were generated with Mercury (version 4.0).^41^ Crystallographic data for the structures reported in this paper have been deposited with the CCDC (2390017 & 2390232†).^42^

### Computational details

Geometry optimizations were performed with the TURBOMOLE software^43^ (v7.2 or v7.5) using the DFT functionals B3LYP^44^ with the def2-TZVPP^45^ basis set, the resolution-of-identity (RI) approximation,^46^ dispersion correction (D3-BJ),^47^ a fine integration grid (m4) and the default SCF convergence criteria (10^−6^ a.u.). All structures were checked for proper spin occupancies and imaginary frequencies with the integrated EIGER and AOFORCE^48^ modules. IR and Raman spectra were simulated at B3LYP(D3BJ)/def2-TZVPP level with a scaling factor of 0.9657,^49^ for transition metal carbonyls, the specialized scaling factor of 0.968 was used.^31^ Gibbs free energies of solvation were calculated with the COSMO-RS model^50^ at the BP86(D3)/def2-TZVPD//BP86(D3)/def-TZVP level of theory using the fine cavity construction algorithm ($cosmo_isorad) and the CosmoThermX software. Relaxed force constants were calculated as diagonal elements of the compliance matrix on the TPSSH/def2-TZVPP level of theory. Evaluating several modern DFT methods the TPSSH functional, additional relying on the electron kinetic energy density, seems to evenly describe the electronic coupling in organometallic compounds.^51^ Transformation of the cartesian DFT force constant matrix into internal coordinates was achieved using the freely available COMPLIANCE 3.0.2. code. Provided that a Cartesian Hessian matrix is available as input, all internal force constants for arbitrary atom–atom pairs, as well as their couplings, can be calculated intuitively using a graphical interface.^52^

### UV-setup

**Fig. 5:**
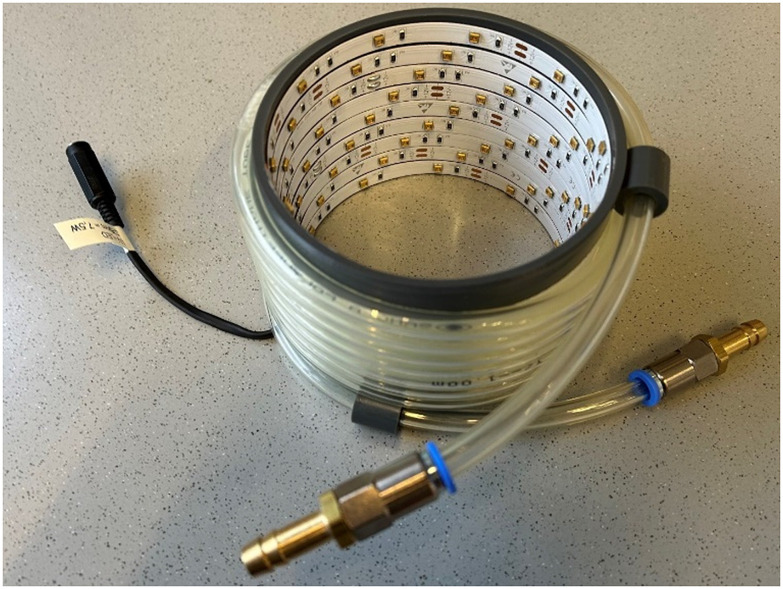
Photograph of the UV-setup.

The Samples were irradiated with a custom-built UV-lamp. A UV-LED strip (370 nm, 47 mW per LED, *ca.* 2.8 m) was glued in a spiral form in a PVC tube with a diameter of 10 cm (Fig. 5). This LED setup has a power of 40 W and a light intensity equivalent to *ca.* 7.5 W. For irradiation durations of over a few minutes, a cooling system is required. This set-up was placed on a stirring plate and the Schlenk-tube inside the lamp, to allow uniform irradiation.

### Synthetic procedures

#### Synthesis of [Ag{Ru(CO)_5_}_2_]^+^[F{Al(OR^F^)_3_}_2_]^−^

Ag^+^[F{Al(OR^F^)_3_}_2_]^−^ (50 mg, 33 μmol, 3.0 eq.) and Ru_3_(CO)_12_ (20 mg, 22 μmol, 2.0 eq.) were filled in a Schlenk tube. 4FB (1 mL) was added to the mixture yielding to a yellow solution. After 20 minutes of irradiation of (7.5 W, 370 nm) UV light, the solution turned colourless. The solution was layered with *n*-pentane (10 mL) and colourless crystals emerged in the following days, which were washed with *n*-pentane (2 × 5 mL) (61 mg, 89%, 29 μmol).

ATR IR (ZnSe) *ṽ*/cm^−1^ = 2162 (vw), 2148 (vw), 2113 (vw), 2098 (sh), 2075 (w), 2067 (w), 1524 (vw), 1508 (vw), 1354 (vw), 1300 (vw), 1276 (w), 1266 (w), 1240 (vs), 1214 (vs), 1177 (w), 1052 (vw), 973 (vs), 863 (vw), 826 (vw), 811 (vw), 760 (vw), 750 (vw), 727 (s), 684 (vw), 637 (vw), 578 (vw), 567 (w).

#### Synthesis of [Ru_2_(CO)_10_]^2+^([Al(OR^F^)_4_]^−^)_2_

[NO]^+^[Al(OC(CF_3_)_3_)_4_]^−^ (100 mg, 0.10 mmol, 3.0 eq.) and 9,10-dichlorooctafluoro-anthracene (40 mg, 0.11 mmol, 3.3 eq.) were filled in a Schlenk tube. 4FB (1 mL) was added to the mixture leading to a colour change and gas evolution. The solution was freeze pumped three times, before Ru_3_(CO)_12_ (20 mg, 0.03 mmol, 1.0 eq.) was added. The reaction was brought to −196 °C using liquid nitrogen and carbon monoxide pressure (3 atm) was added to the tube. After 20 minutes of irradiation of (7.5 W, 370 nm) UV light, the intense green colour of the solution turned to brown indicating a complete reaction of [anthracene^Hal^]˙^+^. The solution was layered with *n*-pentane (10 mL) and colourless crystals emerged in the following days, which were washed with *n*-pentane (2 × 5 mL) (72 mg, 66%, 30 μmol).

ATR IR (ZnSe) *ṽ*/cm^−1^ = 2184 (vw), 2170 (vw), 2150 (vw), 2137 (m), 2105 (vw), 2088 (vw), 1513 (vw), 1447 (vw), 1353 (vw), 1296 (w), 1272 (w), 1264 (w), 1240 (m), 1207 (vs), 1190 (w), 1176 (w), 1126 (vw), 1089 (vw), 1080 (vw), 1068 (vw), 1046 (vw), 967 (vs), 844 (vw), 834 (vw), 756 (vw), 744 (vw), 726 (s), 712 (vw), 682 (vw), 627 (vw), 566 (w), 558 (w).

#### Synthesis of [Ru_2_(CO)_10_]^2+^([F{Al(OR^F^)_3_}_2_]^−^)_2_

The method of [Ru_2_(CO)_10_]^2+^([Al(OR^F^)_4_]^−^)_2_ was repeated, but instead of [NO]^+^[Al(OR^F^)_4_]^−^, [NO]^+^[F{Al(OR^F^)_3_}_2_]^−^ was used.

^1^H-NMR (400 MHz, 4FB, 298 K): only solvent and minor impurities.

^13^C-NMR (50 MHz, 4FB, 298 K): *δ* = 181.0 (s, 8C, [Ru_2_(**C**O)_10_]^2+^ equatorial), 169.3 (s, 2C, [Ru_2_(**C**O)_10_]^2+^ axial), 120.7 (q, 36C, anion – OC(**C**F_3_)_3_), 78.4 (m, 12C, anion – O**C**(CF_3_)_3_) ppm.

^19^F-NMR (188 MHz, 4FB, 298 K): *δ* = −76.2 (s, 108F, anion – OC(C**F**_3_)_3_), −184.8 (s, 2F, anion – Al-**F**-Al) ppm.

## Author contributions

MS performed all the synthetic and analytical work and wrote the manuscript together with IK. JG performed the DFT calculations for the determination of the force constants and added these parts to the manuscript.

## Data availability

The data supporting this article have been included as part of the ESI.†

Crystallographic data for [Ag{Ru(CO)_5_}_2_]^+^[F{Al(OR^F^)_3_}_2_]^−^ and [Ru_2_(CO)_10_]^2+^([F{Al(OR^F^)_3_}_2_]^−^)_2_·(4FB)_2_ has been deposited at the CCDC 2390232 and 2390017.†

## Conflicts of interest

There are no conflicts to declare.

## Supplementary Material

DT-054-D4DT03364G-s001

DT-054-D4DT03364G-s002
