# Supplementary material for: Isolation and characterization of the dimetal decacarbonyl dication [Ru2(CO)10]2+ and the metal-only Lewis-pair [Ag{Ru(CO)5}2]+
Source: Dalton Trans. 2024 Dec 6;54(6):2294–300. doi: 10.1039/d4dt03364g (PMC11640294; doi:10.1039/d4dt03364g)
Supplement: DT-054-D4DT03364G-s001 [file DT-054-D4DT03364G-s001.pdf]

Supplementary Information (SI) for Dalton Transactions.
This journal is © The Royal Society of Chemistry 2024

Supporting Information

to

Isolation and Characterization of the Dimetal Decacarbonyl Dication $[Ru_2(CO)_{10}]^{2+}$ and the Metal-Only Lewis-Pair $[Ag\{Ru(CO)_5\}_2]^+$

by

Malte Sellina, Jörg Grunenbergb and Ingo Krossing*a

Dr. M. Sellin & Prof. Dr. I. Krossing*; Institut für Anorganische und Analytische Chemie und Freiburger Materialforschungszentrum (FMF); Albert-Ludwigs-Universität Freiburg; Albertstr. 21, 79104 Freiburg (Germany). * E-mail: krossing@uni-freiburg.de

Content

IR Spectra 2
NMR Spectra 3
scXRD Data 5
DFT Calculations 6
Coordinates of the DFT-Optimized Structures 7

IR Spectra

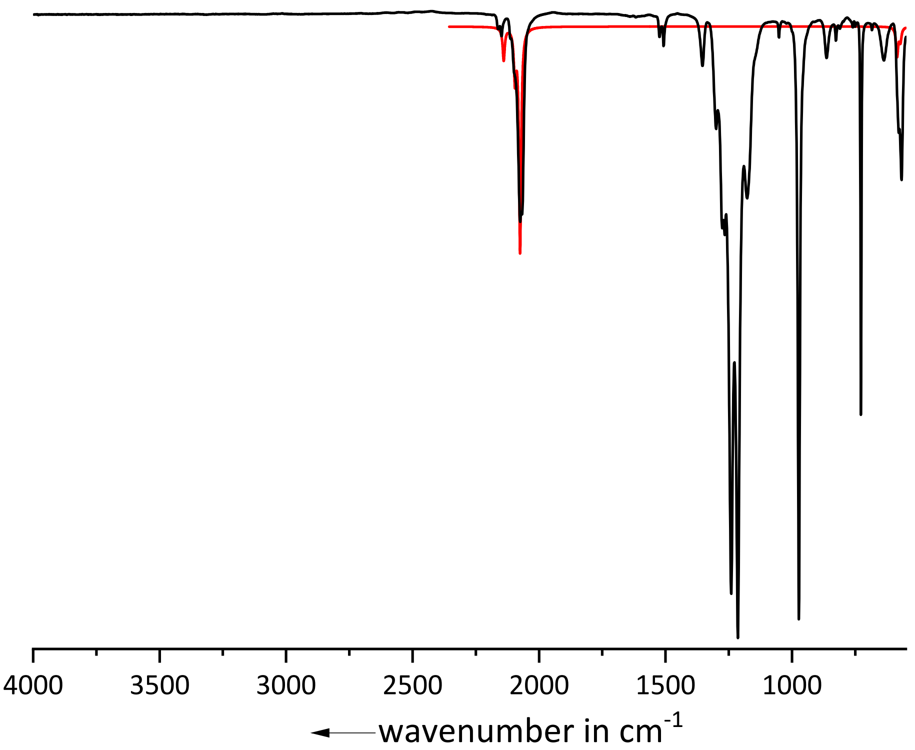

Figure S1: Comparison of the experimental IR spectrum of $[Ag\{Ru(CO)_5\}_2]^+[F\{Al(OR^F)_3\}_2]^-$ (32 scans, black line) with the B3LYP(D3-BJ)/def2-TZVPP calculated spectrum of $[Ag\{Ru(CO)_5\}_2]^+$ (red line) scaled by 0.968 according to Duncan *et al.*

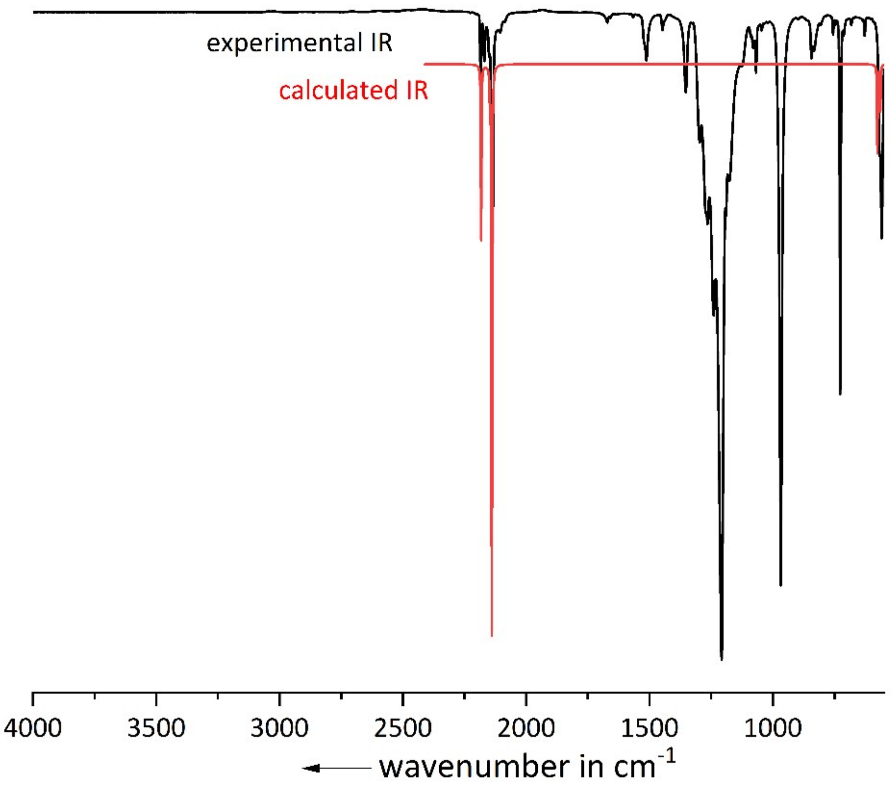

d

Figure S2: Comparison of the experimental IR spectrum of $[Ru_2(CO)_{10}]^{2+}([Al(OR^F)_4]^-)_2$ (32 scans, black line) with the B3LYP(D3-BJ)/def2-TZVPP calculated spectrum of $[Ru_2(CO)_{10}]^{2+}$ (red line) scaled by 0.968 according to Duncan *et al.*

NMR Spectra

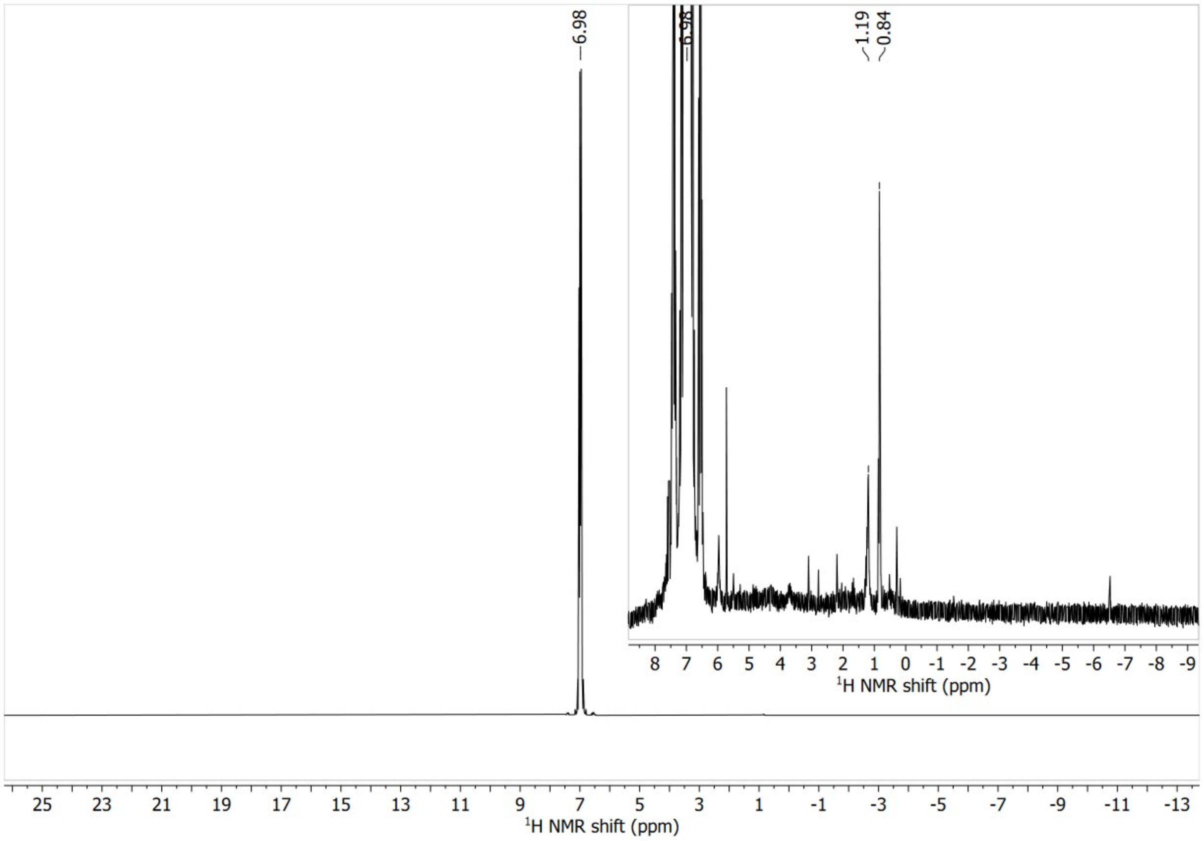

Figure S3: ^{1}H NMR spectrum of $[Ru_2(CO)_{10}]^{2+}([F\{Al(OR^F)_3\}_2]^-)_2$ in 1,2,3,4-tetrafluorobenzene (4FB) at room temperature.

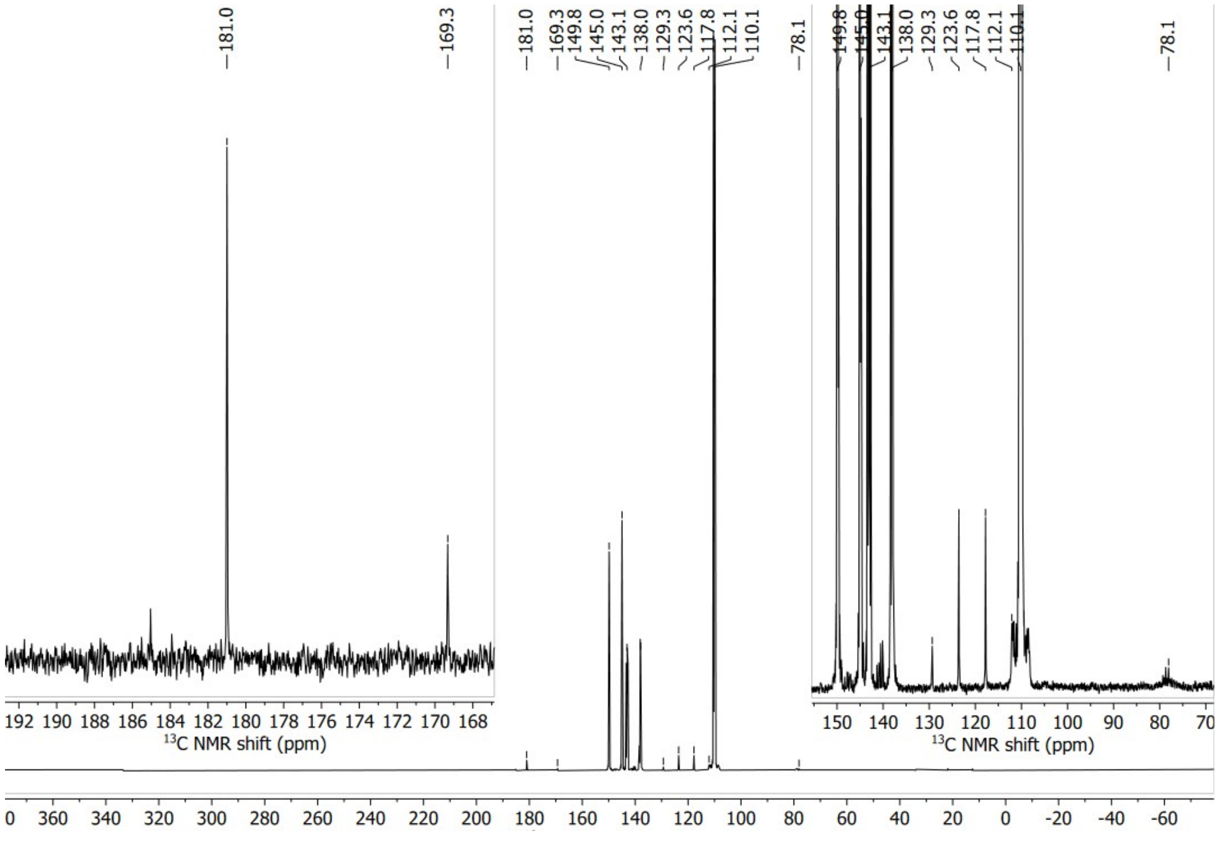

Figure S4: ^{13}C NMR spectrum of $[Ru_2(CO)_{10}]^{2+}([F\{Al(OR^F)_3\}_2]^-)_2$ in 1,2,3,4-tetrafluorobenzene (4FB) at room temperature.

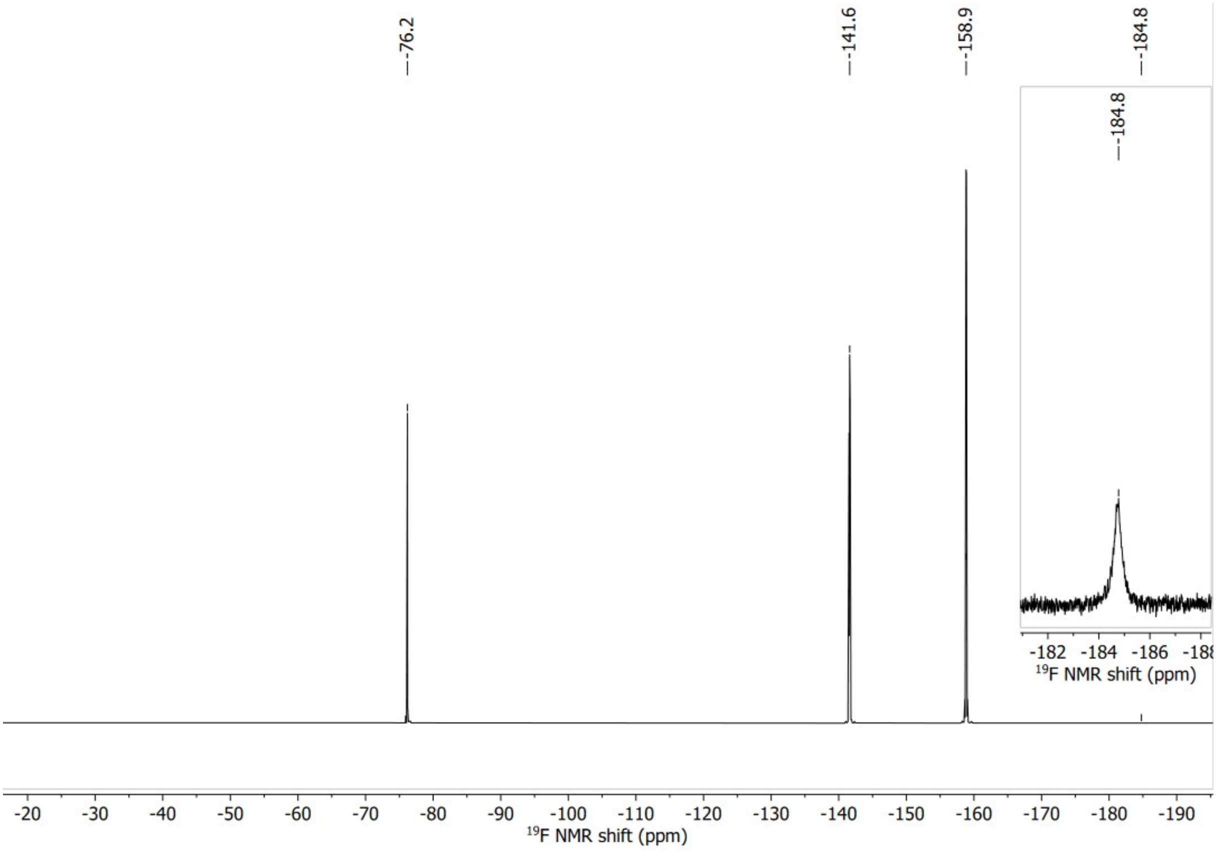

Figure S5: ^{19}F NMR spectrum of $[Ru_2(CO)_{10}]^{2+}([F\{Al(OR^F)_3\}_2]^-)_2$ in 1,2,3,4-tetrafluorobenzene (4FB) at room temperature.

scXRD Data

Table S1: Crystallographic data table for $[Ru_2(CO)_{10}]^{2+}([F\{Al(OR^F)_3\}_2]^-)_2\cdot(4FB)_2$.

CCDC number	2390232	2390017
Empirical formula	$C_{34}AgAl_2F_{55}O_{16}Ru_2$	$C_{70}H_4Al_4F_{118}O_{22}Ru_2$
Formula weight	2073.31	3748.79
Temperature [K]	100(2)	100(2)
Crystal system	triclinic	triclinic
Space group (number)	$P\bar{1}$ (2)	$P\bar{1}$ (2)
a [Å]	11.005(4)	12.594(3)
b [Å]	24.541(7)	19.643(4)
c [Å]	24.767(10)	23.887(6)
α [°]	64.588(17)	90.031(12)
β [°]	84.267(13)	95.653(12)
γ [°]	85.248(13)	90.044(8)
Volume [Å^3]	6005(4)	5880(2)
Z	4	2
ρ_{calc} [gcm^{-3}]	2.293	2.117
μ [mm^{-1}]	1.082	0.537
F(000)	3952	3604
Crystal size [mm^3]	0.220×0.213×0.106	0.249×0.215×0.198
Crystal colour	colourless	colourless
Crystal shape	plate	block
Radiation	MoK_α (λ=0.71073 Å)	MoK_α (λ=0.71073 Å)
2θ range [°]	1.83 to 58.77 (0.72 Å)	3.25 to 56.67 (0.75 Å)
Index ranges	−14 ≤ h ≤ 15<br>−33 ≤ k ≤ 33<br>−33 ≤ l ≤ 33	−16 ≤ h ≤ 16<br>−26 ≤ k ≤ 26<br>−31 ≤ l ≤ 31
Reflections collected	249926	198085
Independent reflections	32365<br>R_{int} = 0.0620<br>R_{sigma} = 0.0389	29301<br>R_{int} = 0.0580<br>R_{sigma} = 0.0354
Completeness to θ = 25.242°	99.5 %	99.9 %
Data / Restraints / Parameters	32365/38094/2496	29301/34962/2424
Absorption correction T_{min}/T_{max} (method)	0.6767/0.7458 (multi-scan)	0.7063/0.7457 (multi-scan)
Goodness-of-fit on F^2	1.139	1.039
Final *R* indexes [*I*≥2σ(*I*)]	R_1 = 0.0836<br>wR_2 = 0.2396	R_1 = 0.0438<br>wR_2 = 0.1011
Final *R* indexes [all data]	R_1 = 0.1033<br>wR_2 = 0.2588	R_1 = 0.0577<br>wR_2 = 0.1101
Largest peak/hole [eÅ^{-3}]	3.68/−2.51[a]	0.81/−1.15

[a] the large residual electron density peaks are near to the ruthenium and silver atom and are likely the result of minor disorders, which could not be resolved.

DFT Calculations

The structures of all particles were calculated at the B3LYP(D3BJ)/def2-TZVPP level of theory. Solvation energies were calculated with COSMO RS (parameterization BP_TZVPD_FINE_22).

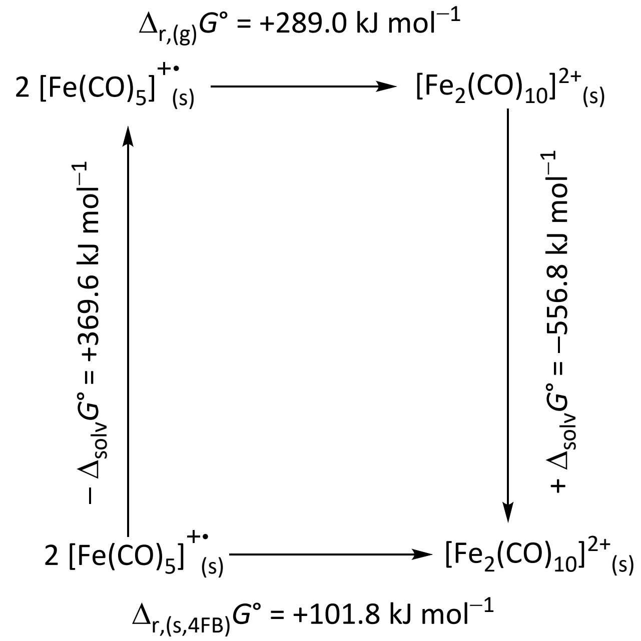

Figure S6: Born-Fajans-Haber cycle of the dimerization of $[Fe(CO)_5]^{+\cdot}$ to $[Fe_2(CO)_{10}]^{2+}$ in 4FB.

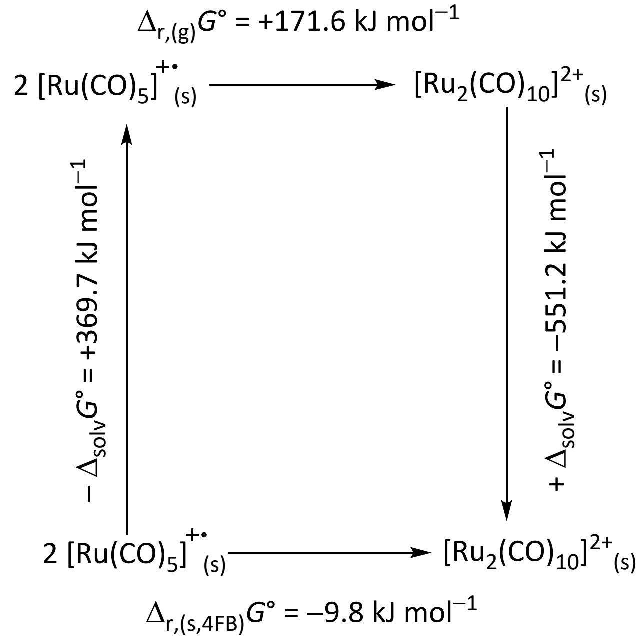

Figure S7: Born-Fajans-Haber cycle of the dimerization of $[Ru(CO)_5]^{+\cdot}$ to $[Ru_2(CO)_{10}]^{2+}$ in 4FB.

Coordinates of the DFT-Optimized Structures

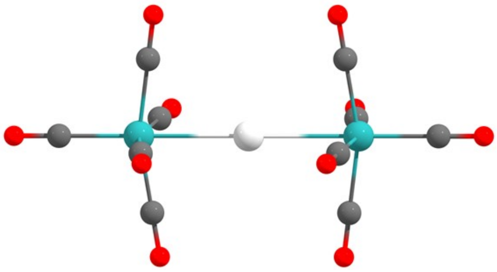

$[Ag\{Ru(CO)_5\}_2]^+$

Method: (RI-)B3LYP(D3BJ)/def2-TZVPP
Symmetry: d4h

Cartesian coordinates in Ångström:

Ag	0.0000000	0.0000000	0.0000000
Ru	-0.0000000	0.0000000	2.7463282
Ru	-0.0000000	0.0000000	-2.7463282
C	-1.9492940	-0.0000000	2.4630625
O	-3.0690890	0.0000000	2.3014785
C	0.0000000	-1.9492940	2.4630625
O	0.0000000	-3.0690890	2.3014785
C	1.9492940	0.0000000	2.4630625
O	3.0690890	0.0000000	2.3014785
C	-0.0000000	1.9492940	2.4630625
O	0.0000000	3.0690890	2.3014785
C	0.0000000	0.0000000	4.7420670
O	0.0000000	0.0000000	5.8703096
C	-1.9492940	0.0000000	-2.4630625
O	-3.0690890	0.0000000	-2.3014785
C	0.0000000	-1.9492940	-2.4630625
O	0.0000000	-3.0690890	-2.3014785
C	1.9492940	0.0000000	-2.4630625
O	3.0690890	0.0000000	-2.3014785
C	-0.0000000	1.9492940	-2.4630625
O	0.0000000	3.0690890	-2.3014785
C	0.0000000	0.0000000	-4.7420670
O	0.0000000	0.0000000	-5.8703096

SCF energy GEOOPT = -1470.159987672 H
ZPE = 220.2 kJ/mol
FREEH energy = 296.76 kJ/mol
FREEH entropy = 0.85713 kJ/mol/K

$vibrational spectrum

# mode	symmetry	wave number	IR intensity	selection rules	
#		cm**(-1)	km/mol	IR	RAMAN
1		-0.00	0.00000	-	-
2		-0.00	0.00000	-	-
3		-0.00	0.00000	-	-
4		-0.00	0.00000	-	-
5		0.00	0.00000	-	-
6		0.00	0.00000	-	-
7	a1u	6.49	0.00000	NO	NO
8	eu	15.26	0.00909	YES	NO
9	eu	15.26	0.00909	YES	NO
10	eg	37.68	0.00000	NO	YES
11	eg	37.68	0.00000	NO	YES
12	b2u	47.17	0.00000	NO	NO
13	b1g	47.25	0.00000	NO	YES
14	eu	61.25	0.19992	YES	NO
15	eu	61.25	0.19992	YES	NO
16	a1g	64.85	0.00000	NO	YES
17	eg	77.51	0.00000	NO	YES
18	eg	77.51	0.00000	NO	YES
19	eu	79.09	0.20797	YES	NO
20	eu	79.09	0.20797	YES	NO
21	b1u	92.01	0.00000	NO	NO
22	b2g	92.07	0.00000	NO	YES

23	eg	94.83	0.00000	NO	YES
24	eg	94.83	0.00000	NO	YES
25	eu	96.23	1.06532	YES	NO
26	eu	96.23	1.06532	YES	NO
27	a2u	100.25	1.09359	YES	NO
28	a1g	127.91	0.00000	NO	YES
29	a2u	175.08	8.27542	YES	NO
30	a1u	351.05	0.00000	NO	NO
31	a2g	351.13	0.00000	NO	NO
32	eg	360.31	0.00000	NO	YES
33	eg	360.31	0.00000	NO	YES
34	eu	361.38	3.26148	YES	NO
35	eu	361.38	3.26148	YES	NO
36	a1g	378.06	0.00000	NO	YES
37	a2u	378.13	44.83253	YES	NO
38	eg	387.17	0.00000	NO	YES
39	eg	387.17	0.00000	NO	YES
40	eu	389.96	64.04219	YES	NO
41	eu	389.96	64.04219	YES	NO
42	b1g	401.53	0.00000	NO	YES
43	b2u	401.70	0.00000	NO	NO
44	b2u	426.76	0.00000	NO	NO
45	b1g	427.52	0.00000	NO	YES
46	a2u	440.20	0.00388	YES	NO
47	a1g	440.91	0.00000	NO	YES
48	eg	478.62	0.00000	NO	YES
49	eg	478.62	0.00000	NO	YES
50	eu	480.03	35.65796	YES	NO
51	eu	480.03	35.65796	YES	NO
52	b1u	506.09	0.00000	NO	NO
53	b2g	507.02	0.00000	NO	YES
54	eg	588.48	0.00000	NO	YES
55	eg	588.48	0.00000	NO	YES
56	eu	590.46	124.62124	YES	NO
57	eu	590.46	124.62124	YES	NO
58	a2u	603.74	529.31866	YES	NO
59	a1g	618.95	0.00000	NO	YES
60	eg	2132.89	0.00000	NO	YES
61	eg	2132.89	0.00000	NO	YES
62	eu	2143.66	2047.63210	YES	NO
63	eu	2143.66	2047.63210	YES	NO
64	b2u	2149.23	0.00000	NO	NO
65	b1g	2155.60	0.00000	NO	YES
66	a2u	2164.62	891.12771	YES	NO
67	a1g	2167.44	0.00000	NO	YES
68	a2u	2210.57	591.77669	YES	NO
69	a1g	2226.51	0.00000	NO	YES

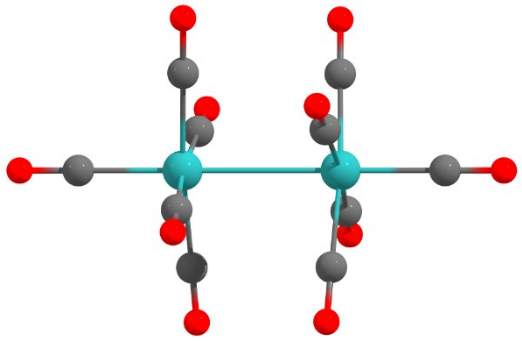

$[Ru_2(CO)_{10}]^{2+}$
Method: (RI-)B3LYP(D3BJ)/def2-TZVPP
Symmetry: d4d

Cartesian coordinates in Ångström:

Ru	-0.0000000	-0.0000000	-1.5074524
Ru	-0.0000000	0.0000000	1.5074524
C	0.7612322	1.8377772	1.4138817
C	0.0000000	-0.0000000	3.5005681
C	1.8377772	-0.7612322	1.4138817
C	-1.8377772	0.7612322	1.4138817
C	-0.7612322	-1.8377772	1.4138817
C	0.7612322	-1.8377772	-1.4138817
C	1.8377772	0.7612322	-1.4138817
C	-1.8377772	-0.7612322	-1.4138817
C	-0.7612322	1.8377772	-1.4138817
C	0.0000000	-0.0000000	-3.5005681
O	1.1907495	2.8747236	1.3665106
O	0.0000000	0.0000000	4.6216200
O	2.8747236	-1.1907495	1.3665106
O	-1.1907495	-2.8747236	1.3665106
O	-2.8747236	1.1907495	1.3665106
O	-2.8747236	-1.1907495	-1.3665106
O	-1.1907495	2.8747236	-1.3665106
O	0.0000000	0.0000000	-4.6216200
O	2.8747236	1.1907495	-1.3665106
O	1.1907495	-2.8747236	-1.3665106

SCF energy GEOOPT = -1322.735888171 H
ZPE = 224.1 kJ/mol
FREEH energy = 293.09 kJ/mol
FREEH entropy = 0.74135 kJ/mol/K

Vibrational spectrum

# mode	symmetry	wave number	IR intensity	selection rules	
#		cm**(-1)	km/mol	IR	RAMAN
1		-0.00	0.00000	-	-
2		-0.00	0.00000	-	-
3		0.00	0.00000	-	-
4		0.00	0.00000	-	-
5		0.00	0.00000	-	-
6		0.00	0.00000	-	-
7	b1	33.77	0.00000	NO	NO
8	e1	55.36	0.06306	YES	NO
9	e1	55.36	0.06306	YES	NO
10	e3	60.53	0.00000	NO	YES
11	e3	60.53	0.00000	NO	YES
12	e2	76.74	0.00000	NO	YES
13	e2	76.74	0.00000	NO	YES
14	e3	82.73	0.00000	NO	YES
15	e3	82.73	0.00000	NO	YES
16	e1	91.45	0.79500	YES	NO
17	e1	91.45	0.79500	YES	NO
18	a1	91.80	0.00000	NO	YES
19	e2	94.31	0.00000	NO	YES

20	e2	94.31	0.00000	NO	YES
21	e1	99.28	0.22526	YES	NO
22	e1	99.28	0.22526	YES	NO
23	e3	102.72	0.00000	NO	YES
24	e3	102.72	0.00000	NO	YES
25	b2	107.00	1.13884	YES	NO
26	a1	141.30	0.00000	NO	YES
27	a2	347.74	0.00000	NO	NO
28	b1	349.76	0.00000	NO	NO
29	e3	351.92	0.00000	NO	YES
30	e3	351.92	0.00000	NO	YES
31	e1	357.40	41.68507	YES	NO
32	e1	357.40	41.68507	YES	NO
33	b2	367.97	0.58052	YES	NO
34	a1	374.12	0.00000	NO	YES
35	e1	386.35	20.28065	YES	NO
36	e1	386.35	20.28065	YES	NO
37	e3	387.79	0.00000	NO	YES
38	e3	387.79	0.00000	NO	YES
39	e2	403.72	0.00000	NO	YES
40	e2	403.72	0.00000	NO	YES
41	b2	412.96	18.38429	YES	NO
42	a1	419.42	0.00000	NO	YES
43	e2	481.58	0.00000	NO	YES
44	e2	481.58	0.00000	NO	YES
45	e3	493.26	0.00000	NO	YES
46	e3	493.26	0.00000	NO	YES
47	e2	499.25	0.00000	NO	YES
48	e2	499.25	0.00000	NO	YES
49	e1	499.99	16.96252	YES	NO
50	e1	499.99	16.96252	YES	NO
51	e3	579.87	0.00000	NO	YES
52	e3	579.87	0.00000	NO	YES
53	e1	582.38	108.78615	YES	NO
54	e1	582.38	108.78615	YES	NO
55	b2	594.99	422.30254	YES	NO
56	a1	613.62	0.00000	NO	YES
57	e3	2193.05	0.00000	NO	YES
58	e3	2193.05	0.00000	NO	YES
59	e1	2210.18	1354.74768	YES	NO
60	e1	2210.18	1354.74768	YES	NO
61	b2	2218.56	114.23267	YES	NO
62	e2	2222.45	0.00000	NO	YES
63	e2	2222.45	0.00000	NO	YES
64	a1	2231.93	0.00000	NO	YES
65	b2	2254.66	833.25172	YES	NO
66	a1	2284.38	0.00000	NO	YES

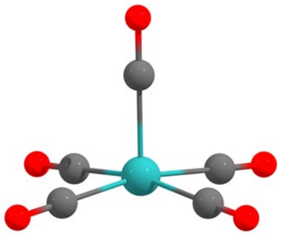

$[Ru(CO)_5]^{+\cdot}$

Method: (RI-)B3LYP(D3BJ)/def2-TZVPP
Symmetry: c4v

Cartesian coordinates in Ångström:

Ru	-0.0000000	0.0000000	0.2869456
C	0.0000000	1.9895393	0.4797727
C	0.0000000	-1.9895393	0.4797727
C	0.0000000	0.0000000	-1.7000016
O	0.0000000	-3.1091301	0.5798222
O	0.0000000	0.0000000	-2.8253236
O	-0.0000000	3.1091301	0.5798222
C	-1.9895393	0.0000000	0.4797727
C	1.9895393	0.0000000	0.4797727
O	3.1091301	0.0000000	0.5798222
O	-3.1091301	-0.0000000	0.5798222

SCF energy GEOOPT = -661.3884273765 H
ZPE = 107.6 kJ/mol
FREEH energy = 140.79 kJ/mol
FREEH entropy = 0.46592 kJ/mol/K

Vibrational spectrum

# mode	symmetry	wave number	IR intensity	selection rules	
#		cm**(-1)	km/mol	IR	RAMAN
1		-0.00	0.00000	-	-
2		-0.00	0.00000	-	-
3		-0.00	0.00000	-	-
4		0.00	0.00000	-	-
5		0.00	0.00000	-	-
6		0.00	0.00000	-	-
7	b1	59.08	0.00000	NO	YES
8	e	77.24	0.05417	YES	YES
9	e	77.24	0.05417	YES	YES
10	b2	90.18	0.00000	NO	YES
11	e	92.70	0.77780	YES	YES
12	e	92.70	0.77780	YES	YES
13	a1	97.93	0.27607	YES	YES
14	e	319.05	1.71216	YES	YES
15	e	319.05	1.71216	YES	YES
16	a2	337.09	0.00000	NO	NO
17	e	351.54	32.47559	YES	YES
18	e	351.54	32.47559	YES	YES
19	a1	352.80	8.53439	YES	YES
20	b1	375.76	0.00000	NO	YES
21	a1	408.20	5.30389	YES	YES
22	b1	435.68	0.00000	NO	YES
23	e	448.57	7.02451	YES	YES
24	e	448.57	7.02451	YES	YES
25	b2	491.96	0.00000	NO	YES
26	e	563.89	60.49025	YES	YES
27	e	563.89	60.49025	YES	YES
28	a1	578.51	73.90819	YES	YES
29	a1	2192.53	465.22906	YES	YES
30	e	2193.40	981.95822	YES	YES
31	e	2193.40	981.95822	YES	YES
32	b1	2209.66	0.00000	NO	YES
33	a1	2263.12	4.94041	YES	YES

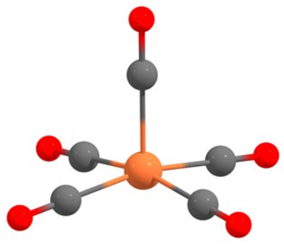

$[Fe(CO)_5]^{+\cdot}$

Method: (RI-)B3LYP(D3BJ)/def2-TZVPP
Symmetry: c4v

Cartesian coordinates in Ångström:

Fe	0.0000000	0.0000000	0.2633004
C	0.0000000	1.8816110	0.4688894
C	0.0000000	-1.8816110	0.4688894
C	-0.0000000	0.0000000	-1.6769927
O	-0.0000000	-2.9983003	0.5872300
O	0.0000000	0.0000000	-2.8002625
O	0.0000000	2.9983003	0.5872300
C	-1.8816110	0.0000000	0.4688894
C	1.8816110	0.0000000	0.4688894
O	2.9983003	-0.0000000	0.5872300
O	-2.9983003	0.0000000	0.5872300

SCF energy GEOOPT = -1830.110841627 H
ZPE = 108.3 kJ/mol
FREEH energy = 141.38 kJ/mol
FREEH entropy = 0.46233 kJ/mol/K

Vibrational spectrum

# mode	symmetry	wave number cm**(-1)	IR intensity km/mol	selection rules IR	RAMAN
1		-0.00	0.00000	-	-
2		-0.00	0.00000	-	-
3		-0.00	0.00000	-	-
4		0.00	0.00000	-	-
5		0.00	0.00000	-	-
6		0.00	0.00000	-	-
7	b1	46.20	0.00000	NO	YES
8	e	77.19	0.04337	YES	YES
9	e	77.19	0.04337	YES	YES
10	b2	95.53	0.00000	NO	YES
11	e	99.54	1.58517	YES	YES
12	e	99.54	1.58517	YES	YES
13	a1	103.75	1.03764	YES	YES
14	e	313.47	0.02162	YES	YES
15	e	313.47	0.02162	YES	YES
16	a1	320.14	1.40158	YES	YES
17	a2	337.97	0.00000	NO	NO
18	b1	344.16	0.00000	NO	YES
19	a1	363.19	6.15358	YES	YES
20	e	390.20	24.54554	YES	YES
21	e	390.20	24.54554	YES	YES
22	b1	417.34	0.00000	NO	YES
23	e	467.56	3.54875	YES	YES
24	e	467.56	3.54875	YES	YES
25	b2	533.90	0.00000	NO	YES
26	a1	572.00	52.81826	YES	YES
27	e	585.45	74.67875	YES	YES
28	e	585.45	74.67875	YES	YES
29	e	2204.48	923.28287	YES	YES
30	e	2204.48	923.28287	YES	YES
31	a1	2209.86	397.77939	YES	YES
32	b1	2219.27	0.00000	NO	YES
33	a1	2263.42	0.85225	YES	YES

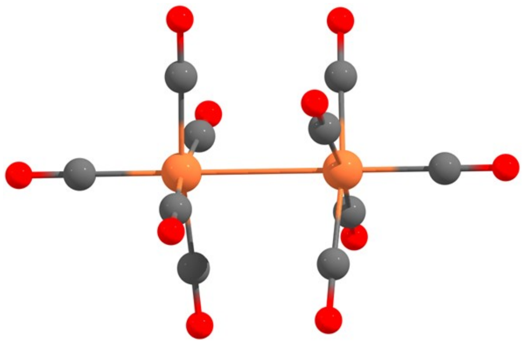

$[Fe_2(CO)_{10}]^{2+}$

```
Method: (RI-)B3LYP(D3BJ)/def2-TZVPP
Symmetry: d4d

Cartesian coordinates in Ångström:
Fe   -0.0000000    0.0000000   -1.4857145
Fe   -0.0000000   -0.0000000    1.4857145
C     0.0000000    0.0000000    3.3644914
C     1.7314490   -0.7171896    1.3797556
C     1.7314490    0.7171896   -1.3797556
O     0.0000000    0.0000000    4.4843576
C     0.0000000    0.0000000   -3.3644914
O     2.7681197    1.1465927   -1.3331588
O     2.7681197   -1.1465927    1.3331588
O     0.0000000    0.0000000   -4.4843576
C    -0.7171896    1.7314490   -1.3797556
C    -0.7171896   -1.7314490    1.3797556
C     0.7171896   -1.7314490   -1.3797556
O    -1.1465927    2.7681197   -1.3331588
C    -1.7314490   -0.7171896   -1.3797556
O    -2.7681197    1.1465927    1.3331588
O    -2.7681197   -1.1465927   -1.3331588
O     1.1465927   -2.7681197   -1.3331588
C     0.7171896    1.7314490    1.3797556
O    -1.1465927   -2.7681197    1.3331588
O     1.1465927    2.7681197    1.3331588
C    -1.7314490    0.7171896    1.3797556

SCF energy GEOOPT =  -3660.138839594 H
ZPE =   227.7 kJ/mol
FREEH energy =   294.98 kJ/mol
FREEH entropy =   0.71755 kJ/mol/K

Vibrational spectrum
#  mode     symmetry     wave number   IR intensity    selection rules
#                         cm**(-1)        km/mol         IR      RAMAN
     1                     -0.00         0.00000         -        -
     2                     -0.00         0.00000         -        -
     3                     -0.00         0.00000         -        -
     4                      0.00         0.00000         -        -
     5                      0.00         0.00000         -        -
     6                      0.00         0.00000         -        -
     7        b1           39.73         0.00000        NO       NO
     8        e1           58.37         0.10998        YES      NO
     9        e1           58.37         0.10998        YES      NO
    10        e3           63.32         0.00000        NO       YES
    11        e3           63.32         0.00000        NO       YES
    12        e2           79.24         0.00000        NO       YES
    13        e2           79.24         0.00000        NO       YES
    14        e3           87.67         0.00000        NO       YES
    15        e3           87.67         0.00000        NO       YES
    16        e1           99.90         1.08582        YES      NO
    17        e1           99.90         1.08582        YES      NO
    18        e2          101.82         0.00000        NO       YES
    19        e2          101.82         0.00000        NO       YES
    20        a1          105.33         0.00000        NO       YES
    21        e1          109.40         0.90835        YES      NO
    22        e1          109.40         0.90835        YES      NO
    23        e3          112.58         0.00000        NO       YES
    24        e3          112.58         0.00000        NO       YES
    25        b2          121.04         0.44163        YES      NO
```

26	a1	211.89	0.00000	NO	YES
27	a2	351.64	0.00000	NO	NO
28	b1	352.86	0.00000	NO	NO
29	b2	358.85	50.34615	YES	NO
30	e3	366.30	0.00000	NO	YES
31	e3	366.30	0.00000	NO	YES
32	e2	373.50	0.00000	NO	YES
33	e2	373.50	0.00000	NO	YES
34	a1	374.83	0.00000	NO	YES
35	e1	379.22	1.06620	YES	NO
36	e1	379.22	1.06620	YES	NO
37	b2	391.46	7.97426	YES	NO
38	a1	411.36	0.00000	NO	YES
39	e1	422.46	28.23573	YES	NO
40	e1	422.46	28.23573	YES	NO
41	e3	427.57	0.00000	NO	YES
42	e3	427.57	0.00000	NO	YES
43	e2	452.61	0.00000	NO	YES
44	e2	452.61	0.00000	NO	YES
45	e3	511.62	0.00000	NO	YES
46	e3	511.62	0.00000	NO	YES
47	e1	524.35	7.33156	YES	NO
48	e1	524.35	7.33156	YES	NO
49	e2	542.95	0.00000	NO	YES
50	e2	542.95	0.00000	NO	YES
51	e3	605.46	0.00000	NO	YES
52	e3	605.46	0.00000	NO	YES
53	b2	607.67	440.08946	YES	NO
54	e1	607.85	140.60405	YES	NO
55	e1	607.85	140.60405	YES	NO
56	a1	636.07	0.00000	NO	YES
57	e3	2201.11	0.00000	NO	YES
58	e3	2201.11	0.00000	NO	YES
59	b2	2211.02	162.08006	YES	NO
60	e1	2218.09	1222.51487	YES	NO
61	e1	2218.09	1222.51487	YES	NO
62	e2	2219.61	0.00000	NO	YES
63	e2	2219.61	0.00000	NO	YES
64	a1	2240.84	0.00000	NO	YES
65	b2	2249.43	1082.87092	YES	NO
66	a1	2277.52	0.00000	NO	YES

$end
